# Mapping of disease-specific Oxford Knee Score onto EQ-5D-5L utility index in knee osteoarthritis

**DOI:** 10.1186/s13018-023-03522-0

**Published:** 2023-02-02

**Authors:** Hadeer Fawaz, Omaima Yassine, Abdullah Hammad, Ramez Bedwani, Ghada Abu-Sheasha

**Affiliations:** 1 Department of Biomedical Informatics and Medical Statistics, Medical Research Institute, University of Alexandria, 165, Horreya Avenue, Hadara, Alexandria, Egypt; 2 Department of Orthopaedic Surgery and Traumatology, El‑Hadra Hospital, University of Alexandria, Alexandria, Egypt

**Keywords:** Model mapping, EQ-5D-5L, Quality of life utility index, Oxford Knee Score (OKS) questionnaire

## Abstract

**Background:**

EQ5D is a generic measure of health. It provides a single index value for health status that can be used in the clinical and economic evaluation of healthcare. Oxford Knee Score (OKS) is a joint-specific outcome measure tool designed to assess symptoms and function in osteoarthritis patients after joint replacement surgery. Though widely used, it has the disadvantage of lacking health index value. To fill the gap between functional and generic questionnaires with economic value, we linked generic EQ-5D-5L to the specific OKS to give a single index value for health status in KOA patients.

**Questions/purposes:**

Developing and evaluating an algorithm to estimate EuroQoL generic health utility scores (EQ-5D-5L) from the disease-specific OKS using data from patients with knee osteoarthritis (KO).

**Patients and methods:**

This is a cross-sectional study of 571 patients with KO. We used four distinct mapping algorithms: Cumulative Probability for Ordinal Data, Penalized Ordinal Regression, CART (Classification and Regression Trees), and Ordinal random forest. We compared the resultant models’ degrees of accuracy.

**Results:**

Mobility was best predicted by penalized regression with pre-processed predictors, usual activities by random forest, pain/discomfort by cumulative probability with pre-processed predictors, self-care by random forest with RFE (recursive feature elimination) predictors, and anxiety/depression by CART with RFE predictors. Model accuracy was lowest with anxiety/depression and highest with mobility and usual activities. Using available country value sets, the average MAE was 0.098 ± 0.022, ranging from 0.063 to 0.142; and the average MSE was 0.020 ± 0.008 ranging from 0.008 to 0.042.

**Conclusions:**

The current study derived accurate mapping techniques from OKS to the domains of EQ-5D-5L, allowing for the computation of QALYs in economic evaluations. A machine learning-based strategy offers a viable mapping alternative that merits further exploration.

**Supplementary Information:**

The online version contains supplementary material available at 10.1186/s13018-023-03522-0.

## Introduction

Osteoarthritis (OA) is a chronic disease that affects the knee joint. The lifetime risk of knee osteoarthritis (KOA) is approximately 46%. Globally, 85% of the burden of osteoarthritis is attributable to KOA [2], making it the eleventh contributor to global disability and the 38th in terms of disability-adjusted life years (DALYs) [3].

Osteoarthritis impacts every part of daily life. Associated deformity results in a rigid, unstable, and painful gait that reduces the independent walking distance and is accompanied by weight gain, sleep problems, and depression [4].

KOA induces substantial costs. With the ageing of the population and the increasing obesity in many countries, the economic burden on healthcare systems could be even higher in the coming years [36]. This requires decision-makers to rely on economic evaluations for optimal resource allocation and maximizing health benefits from fixed budgets.

In economic evaluations, generic questionnaires are preferable to disease-specific questionnaires in order to compare the value of interventions across various disease areas and programs. Most of the official national pharmacoeconomic evaluation guidelines mention EQ-5D by name as a preferred instrument for the determination of health utilities or as an illustration of a suitable instrument. Of those that did not specify a specific measure, the majority of guidelines favored calculating utilities using national preference weights, which are generally derived from societal preferences for health states [29].

However, the Oxford knee score (OKS) was used in the majority of published studies evaluating the interventions used to treat KOA; this score has the drawbacks of being disease-specific and lacking a preference-based index value [26].

Mapping can offer a solution when EQ-5D scores are unavailable for interventions of interest, where health-related utilities can be generated from another measure of health outcomes [5]. By mapping OKS scores to EQ-5D, we can use results from OKS-based previous studies without having to re-run them using EQ-5D questionnaires.

Two strategies are used in mapping studies: direct utility mapping and indirect response mapping. The EQ-5D index value (utility) is predicted using direct mapping, whereas the responses to EQ-five domains are predicted using response mapping. Although response mapping requires an additional step to estimate the expected index value using available EQ-5D tariffs, indirect mapping allows for the prediction of EQ-5D-5L utility values for any country. Direct mapping would only be applicable for the country that produces the tariff [19].

### Aim of the work

This study aims to develop indirect mapping algorithms that can predict responses to the five domains of EQ-5D based on OKS values. Utility values can be then derived from the predicted responses as a separate second step using available EQ-5D tariffs.

## Material and methods

### Included patients

Adults over 18 years old with KOA based on clinical and radiographic characteristics, with or without total knee arthroplasty (TKA), were included in the samples. Patients whose conditions prevented them from completing the questionnaires were excluded (e.g., severe organic or psychiatric diseases). The Institutional Review Board of Medical Research Institute has granted ethical approval following U.S. Department of Health and Human Services (IORG 0008812) guidelines and other applicable regulations. The research adhered to the Declaration of Helsinki's principles.

Two cross-sectional samples were collected: estimation and external validation samples. For the estimation sample, 456 (80% of the whole sample) were recruited between December 2020 and May 2021 and used to develop the model. From September to October 2021, the external validation sample (*n* = 115) was collected to assess the generalizability of the developed model. By recruiting patients at different times, we aimed to have structurally different samples [28]. Justice AC Suggested evaluating the generalizability of a model using data unavailable at the time of model development. When the external validation sample closely resembles the estimation sample, the evaluation focuses on reproducibility rather than generalizability.

Using a self-administered questionnaire, the following data was gathered:

1. Patient characteristics: sex, age, weight, height, duration of OA, presence of TKA, and co-morbidities.
2. OKS questionnaire (12 questions). Each response level ranges between 0 and 4. The ratings ranged from 0 to 48, with 48 representing the best health (7,8). In Egypt, the questionnaire was translated and validated. (9) The score was classified as very mild (40 to 48), mild (30 to 39), moderate (20 to 29), and severe (0–19) [6] .
3. The EQ-5D-5L questionnaire evaluates health status in five domains: mobility, self-care, routine activities, pain/discomfort, and anxiety/depression. Each response level ranges between 1 and 5. In addition, the patient is required to record their overall health status using a visual analogue scale (EQ-VAS). Following assessment, the scores from the descriptive component can be reported as a five-digit number, known as profile scores. There are a total of 3,125 profile scores for EQ-5D-5L, ranging from 11,111 (full health) to 55,555 (worst health). The profile score can be converted into a utility index using a country-specific value set. Two types of value sets are available for many countries: valuation value sets, which were generated using a time trade-off (cTTO) valuation technique supplemented by a discrete choice experiment (DCE), and cross-walk value sets, which were generated by mapping between the EQ-5D-5L and EQ-5D-3L descriptive systems [21]. All countries' value sets were obtained from the EuroQol.org website [7].

### Statistical analysis

#### Conceptual overlap

Spearman's rank correlation was used to determine the conceptual overlap between the domains of EQ-5D-5L and the 12 questions of OKS. The similarity between the two measurements was examined using an exploratory Ordinary Least Squares (OLS) model, where the dependent variable was EQ-5D-5L, and the regressor was the total OKS score.

**Fig. 1:**
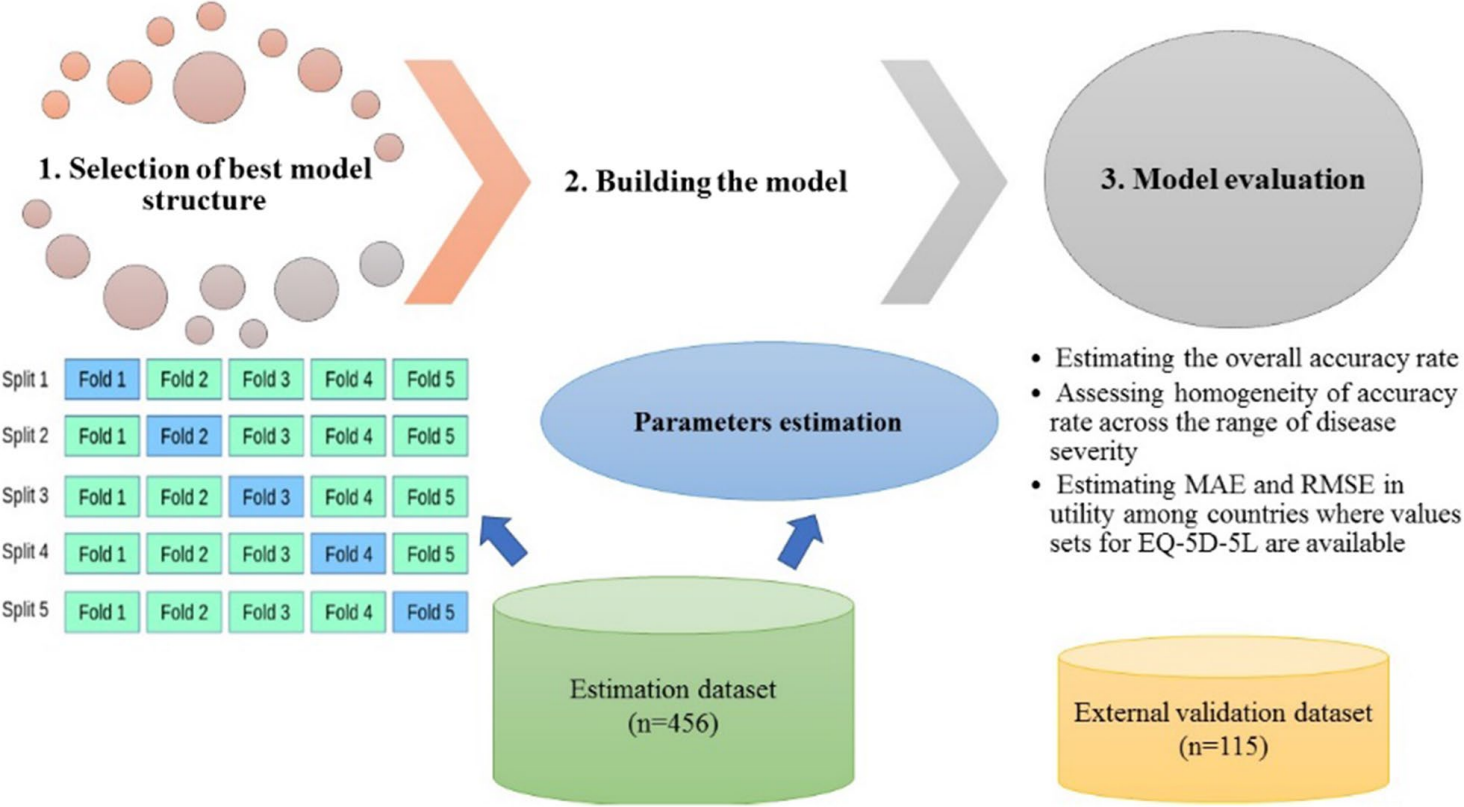
Summarizes the methods used for model selection, building, and evaluation

Method of model selection, building, and evaluation (Fig. 1).

#### Selection of the optimum model structure

Four classes of ordinal models were evaluated: two regression models (cumulative model and penalized ordinal regression) and two tree-based models (ordinal classification and regression trees (O-CART) and ordinal forests (OF)). The binomial and multinomial models were ruled out because they disregard information about the outcome's order. Each model class can accept distinct structures with varying performance. The structure of a model is determined by its hyperparameters and their values. Box 1 describes the model classes and their hyperparameters in detail.

Which predictors are incorporated into the model, and in what form have a substantial impact on its predictive performance. Consequently, each model structure was constructed using four distinct sets of predictors (all derived from OKS questions) (1) all predictors; (2) REF-based significant predictors; (3) model-based significant predictors; and (4) principal components. Box 2 provides a summary of how sets were identified.

We employed 5 × threefold cross-validations to determine the optimal model structure based on model trials. The estimation sample was divided into five non-overlapping folds, with each fold serving as an internal validation set to assess the accuracy of the model developed using the other four folds. This was repeated three times for every model structure and predictor set. Each model's cross-validated accuracy rate was the mean of the 5 × threefold accuracy rates. The optimal model structure was the one with the highest accuracy rate when cross-validated.

#### Model building

After identifying the optimum model structure and best set of predictors in the previous step, the whole estimation sample was used to estimate the model parameters. Model parameters specify how to calculate the outcome from the predictors. They are estimated by optimizing the model's fitness for the estimation sample (Table 1).

**Table 1:** Summary for the structure and number of models tried to build a mapping algorithm from OKS to each of the five domains of EQ-5D-5L

Model class	Hyperparameters			Model structures (*n*)^a^	Model trials (*n*)^b^
	Hyperparameter	Values	Number		
Cumulative model (CM)	Parallelism	TRUE or FALSE	2	10	40
	Link	Logit, Probit, Cauchit, Cloglog, or Logc	5		
Penalized regression	Alpha	Ridge or Lasso	2	16	64
	Criteria	AIC or BIC	2		
	Link	Logit, Probit, Cauchit, or Cloglog,	4		
Ordinal CART	CP	20 randomly selected values	20	80	320
	Split	Misclassification cost in absolute or quadratic terms	2		
	Prune	Misclassification rate or cost	2		
Ordinal forest	Nsets	50, 100, or 150	3	27	108
	Ntreeperdiv	50, 100, or 150	3		
	Ntreefinal	200, 400, or 600	3		
				133	532

^a^Refers to the number of model structures per every model class. It is the product of multiplication of the number of the values taken by each hyperparameter, e.g., in the cumulative model, Parallelism and Link can take two and 5 different values respectively, resulting in 10 different CM structures

^b^Refers to the number of model trials per every model class. It is the product of multiplication of the number of structures and the number of tried sets of predictors (4 sets). Link, link function used to transform cumulative probability into an unbounded scale; *α*, the term of penalty; criteria, criteria used for selecting the optimum magnitude of penalty; CP, Complexity Parameter, Split, criteria for splitting; Prune: criteria for pruning; nsets, number of score sets tried before the approximation of the optimal score set; ntreeperdiv, number of trees in the smaller forests; ntreefinal, number of trees in the final OF constructed using the optimized score set

#### Model evaluation

Evaluation of the predictive performance of the top models was conducted as follows:

1. Comparing the accuracy of no model (baseline accuracy) to the accuracy of the best model.
  The base level of precision for each domain is the proportion of the most prevalent level. [8]. The crude accuracy attained by the final model for each domain is the proportion of accurate predictions made on estimation and external validation samples[9].
2. Estimating the performance of models in terms of errors in measuring predicted utility values
  The levels in the five domains were combined to determine both the actual and predicted profile scores. Actual and predicted utilities were estimated using available tariffs (*n*=39) and eq5d R package [14], For each value set, the mean absolute error (MAE) and mean squared error (MSE) for differences between observed and predicted EQ-5D-5L index scores were calculated. Using the boot package, the 95% confidence interval for these measurements was calculated [15].

Comparing the MAE between utilities above and below the median estimated utility to assess the model's ability to fit patients with better and worse estimated utilities. We followed the Mapping onto Preference-based Measures Reporting Standards (MAPS) statement to improve the clarity, transparency, and thoroughness of mapping study reporting [33].

Model building and evaluation were conducted using caret R package [10].

## Results

### General characteristics

#### The estimation sample

The estimation sample had a mean age of 47.6 ± 13.3 years, and 321 (70.4%) were female. OA lasted an average of 6.7 ± 6.5 years. About 26% of patients complained of back pain, with hypertension being the most common comorbidity (Additional file 1). Approximately 13.4% had undergone TKR, 24.1% were indicated for TKR, and 62.5% were not. Level 1 was the most frequently reported level for mobility (27%) and self-care (60%), level 2 for typical activities (25%), levels 2 and 3 for pain/discomfort (30%), and level 3 for anxiety/depression (30%). The average EQ-VAS was 61.2 ± 24.7

The estimation sample expressed 206 of the 3125 EQ-5D-5L health conditions, with utilities ranging from − 0.964 to 1 (Additional file 1). Maximum and minimum utility indices were reported by equal numbers (3.1% and 2.2%, respectively) (Table 2).

**Table 2:** Description of EQ-5D-5L domains and VAS as well as OKS in the Estimation sample

Question/Score	Whole estimation sample		TKR					
			No				Yes	
			Not indicated		Indicated			
	(*n* = 456)		(*n* = 285)		(*n* = 110)		(*n* = 61)	
Mobility								
L1	121	26.5%	102	35.8%	2	1.8%	17	27.9%
L2	111	24.3%	93	32.6%	3	2.7%	15	24.6%
L3	103	22.6%	80	28.1%	15	13.6%	8	13.1%
L4	94	20.6%	10	3.5%	66	60%	18	29.5%
L5	27	5.9%	–	0%	24	21.8%	3	4.9%
Self-care								
L1	271	59.4%	219	76.8%	16	14.5%	36	59%
L2	61	13.4%	44	15.4%	10	9.1%	7	11.5%
L3	62	13.6%	21	7.4%	34	30.9%	7	11.5%
L4	38	8.3%	–	0%	31	28.2%	7	11.5%
L5	24	5.3%	1	0.4%	19	17.3%	4	6.6%
Usual activities								
L1	112	24.6%	97	34%	1	0.9%	14	23%
L2	116	25.4%	97	34%	4	3.6%	15	24.6%
L3	113	24.8%	80	28.1%	20	18.2%	13	21.3%
L4	68	14.9%	10	3.5%	47	42.7%	11	18%
L5	47	10.3%	1	0.4%	38	34.5%	8	13.1%
Pain/discomfort								
L1	38	8.3%	30	10.5%	1	0.9%	7	11.5%
L2	140	30.7%	120	42.1%	2	1.8%	18	29.5%
L3	140	30.7%	111	38.9%	15	13.6%	14	23%
L4	77	16.9%	20	7.0%	45	40.9%	12	19.7%
L5	61	13.4%	4	1.4%	47	42.7%	10	16.4%
Anxiety/depression								
L1	118	25.9%	86	30.2%	10	9.1%	22	36.1%
L2	114	25%	89	31.2%	11	10%	14	23%
L3	138	30.3%	87	30.5%	40	36.4%	11	18%
L4	48	10.5%	16	5.6%	23	20.9%	9	14.8%
L5	38	8.3%	7	2.5%	26	23.6%	5	8.2%
EQ-5D VAS^€^	61.2 ± 24.7		69.8 ± 17.3		38.5 ± 25.8		59.6 ± 27.5	
Egypt utility	0.38 ± 0.53		0.65 ± 0.26		− 0.30 ± 0.40		0.35 ± 0.56	
Total OKS	27.3 ± 13.2		34.3 ± 7.46		10.1 ± 5.75		25.3 ± 14.5	
Usual level of pain	1.11 ± 1.11		1.44 ± 1.04		0.20 ± 0.57		1.18 ± 1.26	
Trouble with washing and drying	3.06 ± 1.28		3.66 ± 0.72		1.56 ± 1.10		2.95 ± 1.40	
Trouble with transport	2.39 ± 1.33		3.01 ± 0.96		0.91 ± 0.80		2.20 ± 1.47	
Walking time before severe pain	2.70 ± 1.23		3.29 ± 0.79		1.36 ± 1.00		2.34 ± 1.29	
Pain on standing up from sitting	2.16 ± 1.19		2.61 ± 0.98		1.00 ± 0.77		2.13 ± 1.26	
Limping	2.38 ± 1.47		3.06 ± 0.98		0.76 ± 0.98		2.10 ± 1.71	
Difficulty kneeling	2.11 ± 1.42		2.59 ± 1.23		0.94 ± 1.1		1.95 ± 1.53	
Pain at night	2.16 ± 1.45		2.80 ± 1.15		0.60 ± 0.74		2.03 ± 1.51	
Pain interferes with work	2.12 ± 1.36		2.75 ± 0.98		0.53 ± 0.63		2.03 ± 1.47	
Sense of knee instability	2.44 ± 1.43		3.14 ± 0.89		0.77 ± 0.93		2.20 ± 1.63	
Can do household shopping alone	2.66 ± 1.53		3.48 ± 0.88		0.76 ± 0.95		2.26 ± 1.60	
Trouble walking downstairs	1.98 ± 1.25		2.49 ± 1.00		0.66 ± 0.63		1.95 ± 1.41	

Values are number and % or mean ± SD

€ missing cases in vas are 18 (3.9%)

OKS is Oxford knee score, each question ranges (0–4), (0) means severe, (4) means very mild

L level of the domain of EQ-5D-5L, each domain ranges (1–5), (1) means no problems, (5) means unable to / extreme problems

VAS is a visual analogue scale, it ranges (0–100), (0) means the worst health you can imagine, (100) means the best health you can imagine

Egypt utility ranges from − 0.964 for the worst health state (55555) to 1 for full health (11111)

% are estimated from columns

#### The external validation sample

The external validation sample had a mean age of 49 ± 14.1 years, and 78 (80.9%) were female. On average, osteoarthritis lasted 6.7 ± 6.5 years. About 28% of patients complained of back pain, with hypertension being the most common comorbidity (Additional file 1). Approximately 24.3% had undergone TKR, 18.3% were indicated for TKR, and 57.4% were not.

Level 1 was the most frequently reported level for mobility (39%), self-care (57%), usual activities (30%) and anxiety/depression (38.3%), and level 3 for pain/discomfort (33%). The average EQ-VAS was 69.3 ± 21.3.

They expressed 67 different EQ-5D-5L health conditions, with utilities ranging from − 0.732 to 1 (Additional file 1). Maximum and minimum utility indices were reported by 10.4% and 0.9%, respectively (Table 3).

**Table 3:** Description of EQ-5D-5L domains and VAS as well as OKS in the external validation sample

	Whole external validation sample		TKR					
			No				Yes	
			Not indicated		Indicated			
	(*n* = 115)		(*n* = 66)		(*n* = 21)		(*n* = 28)	
Mobility								
L1	45	39.1%	31	47%	–	0%	14	50%
L2	18	15.7%	14	21.2%	–	0%	4	14.3%
L3	30	26.1%	18	27.3%	4	19.0%	8	28.6%
L4	17	14.8%	3	4.5%	12	57.1%	2	7.1%
L5	5	4.3%	–	0%	5	23.8%	–	0%
Self-care								
L1	66	57.4%	49	74.2%	–	0%	17	60.7%
L2	23	20%	12	18.2%	5	23.8%	6	21.4%
L3	12	10.4%	4	6.1%	5	23.8%	3	10.7%
L4	11	9.6%	1	1.5%	8	38.1%	2	7.1%
L5	3	2.6%	–	0%	3	14.3%	–	0%
Usual activities								
L1	35	30.4%	24	36.4%	–	0%	11	39.3%
L2	31	27%	23	34.8%	–	0%	8	28.6%
L3	28	24.3%	17	25.8%	5	23.8%	6	21.4%
L4	14	12.2%	2	3.0%	10	47.6%	2	7.1%
L5	7	6.1%	–	0%	6	28.6%	1	3.6%
Pain/discomfort								
L1	19	16.5%	9	13.6%	–	0%	10	35.7%
L2	37	32.2%	29	43.9%	–	0%	8	28.6%
L3	38	33%	24	36.4%	5	23.8%	9	32.1%
L4	11	9.6%	2	3%	9	42.9%	–	0%
L5	10	8.7%	2	3%	7	33.3%	1	3.6%
Anxiety/depression								
L1	44	38.3%	24	36.4%	3	14.3%	17	60.7%
L2	35	30.4%	22	33.3%	5	23.8%	8	28.6%
L3	28	24.3%	18	27.3%	9	42.9%	1	3.6%
L4	6	5.2%	1	1.5%	3	14.3%	2	7.1%
L5	2	1.7%	1	1.5%	1	4.8%	–	0%
EQ-5D VAS	69.3 ± 21.3		74.3 ± 17.8		47.4 ± 19.9		73.9 ± 19.9	
Egypt utility	0.52 ± 0.47		0.68 ± 0.24		− 0.22 ± 0.37		0.69 ± 0.41	
Total OKS	30.6 ± 12.4		34.8 ± 7.83		10.8 ± 5.63		35.5 ± 10.3	
Usual level of pain	1.50 ± 1.40		1.45 ± 1.17		0.095 ± 0.3		2.68 ± 1.39	
Trouble with washing and drying	3.19 ± 1.19		3.67 ± 0.69		1.33 ± 0.97		3.46 ± 0.92	
Trouble with transport	2.56 ± 1.18		2.92 ± 0.90		1.10 ± 0.94		2.79 ± 1.10	
Walking time before severe pain	2.93 ± 1.13		3.52 ± 0.75		1.43 ± 0.75		2.68 ± 0.98	
Pain on standing up from sitting	2.48 ± 1.19		2.64 ± 1.00		1.00 ± 0.71		3.21 ± 0.96	
Limping	2.83 ± 1.25		3.18 ± 0.89		1.19 ± 0.99		3.21 ± 1.20	
Difficulty kneeling	2.08 ± 1.49		2.38 ± 1.37		0.57 ± 0.87		2.50 ± 1.45	
Pain at night	2.73 ± 1.33		3.06 ± 1.16		1.00 ± 0.89		3.25 ± 0.84	
Pain interferes with work	2.39 ± 1.30		2.68 ± 1.13		0.72 ± 0.46		2.96 ± 1.07	
Sense of knee instability	2.80 ± 1.26		3.30 ± 0.82		0.95 ± 0.86		3.00 ± 1.12	
Can do household shopping alone	2.94 ± 1.37		3.55 ± 0.89		0.95 ± 1.20		3.00 ± 0.99	
Trouble walking downstairs	2.16 ± 1.28		2.45 ± 1.04		0.48 ± 0.75		2.71 ± 1.08	

Values are number and % or mean ± SD

OKS is Oxford knee score, each question ranges (0–4), (0) means severe, (4) means very mild

L level of the domain of EQ-5D-5L, each domain ranges (1–5), (1) means no problems, (5) means unable to / extreme problems

VAS is a visual analogue scale, it ranges (0–100), (0) means the worst health you can imagine, (100) means the best health you can imagine

Egypt utility ranges from − 0.964 for the worst health state (55555) to 1 for full health (11111)

% are estimated from columns

### Exploratory data analysis

#### Conceptual overlap

The significant correlations between EQ-5D-5L domains and OKS questions ranged from − 0.79 to − 0.28 (Additional file 1). The prevalence of blue hues throughout the plot indicates a robust first principal component, which accounts for 66.35 per cent of the total variance.

#### Important questions as determined by recursive feature elimination (RFE)

RFE ranks predictors based on their contribution to every domain (Additional file 1). All questions (*n* = 12) contributed to mobility. Eleven, eight, and seven questions pertained to usual activities, pain/discomfort, and self-care, respectively. Only three questions contributed to anxiety/depression.

"Walking time before severe pain" was the first contributing question in predicting mobility. “Troubles with washing and drying" topped the self-care list, and "Pain interferes with work" topped the lists for usual activities, pain/discomfort, and anxiety/depression.

#### Model building on the estimation sample

After constructing models, we compared and selected the most accurate model for each domain (Table 4) (Additional file 1). Cross-validation accuracy was highest for self-care and lowest for anxiety/depression. Cross-validation yielded coefficients of variation of 5% for self-care, 6% for mobility and pain/discomfort, 7% for usual activity, and 9% for anxiety/depression.

**Table 4:** Measures of performance (accuracy) of the best models in the five domains on the estimation and external validation sample

Domain	Model type	Preparation of predictors	Tuned hyperparameters	Accuracy			
				Estimation sample			External validation sample
				Baseline^1^	Crude^2^	CV^3^	Crude^2^
				(95% CI)	(95% CI)	(SD)	(95% CI)
MO	Penalized regression	Pre-processed	alpha = 1	26.5% (22.6,.30.9)	0.658 (0.612, 0.701)	0.656	0.687
			criteria = aic			(0.037)	(0.593, 0.770)
			link = cauchit				
SC	Random forest	RFE	nsets = 150	59.4% (54.7, 63.9)	0.840 (0.803, 0.872)	0.724	0.669
			ntreeperdiv = 150			(0.039)	(0.575, 0.754)
			ntreefinal = 600				
UA	Random forest	All predictors	nsets = 50	25.4% (21.5, 29.7)	0.882 (0.848, 0.91)	0.604	0.687
			ntreeperdiv = 100			(0.044)	(0.593, 0.770)
			ntreefinal = 200				
PD	Cumulative probability model	Pre-processed	parallel = TRUE	30.7% (26.5, 35.2)	0.686 (0.642, 0.729)	0.671	0.678
			link = cauchit			(0.039)	(0.584, 0.762)
AD	CART	RFE	cp = 0.00645	30.3% (26.1, 34.7)	0.452 (0.405, 0.499)	0.435	0.357
			split = abs			(0.038)	(0.269, 0.451)
			prune = mc				

^1^*Baseline accuracy* in each domain is the proportion of the most common level

^2^*The crude accuracy* is the proportion of all correct predictions

^3^*The cross-validated accuracy rate* was the average of the 5 × 3-folds accuracy rates conducted on the estimation sample. MO is mobility, SC self-care, UA usual activities, PD pain/discomfort, and AD anxiety/depression. Pre-processed predictors extracted using PCA; alpha (α), the term of penalty; criteria, criteria used for selecting the optimum magnitude of penalty; Link, link function used to transform cumulative probability into an unbounded scale; RFE, recursive feature elimination; nsets, number of score sets tried before the approximation of the optimal score set; ntreeperdiv, number of trees in the smaller forests; ntreefinal, number of trees in the final OF constructed using the optimized score set; parallel, parallel curves or not; CP, complexity parameter; Split, criteria for splitting; Prune, criteria for pruning

#### Model evaluation on the external validation sample

In the external validation sample, the performance of the models predicting all domains yielded greater crude accuracy than the baseline accuracy (Table 4). The mobility domain's predictive accuracy increased from 26.5% (baseline accuracy) to 65.6% in the estimation sample and to 68.2% in the external validation sample. The models' accuracy was lowest for anxiety/depression and highest for mobility and usual activity.

The five EQ-5D domains were predicted using the models with the highest accuracies. Mobility was predicted by penalized regression with pre-processed predictors, usual activities by random forest, pain/discomfort by cumulative probability with pre-processed predictors, self-care by random forest with RFE predictors, and anxiety/depression by CART with RFE predictors.

Actual and predicted EQ-5D-5L utility values were estimated for all countries with available tariffs (either valuation technique VT or crosswalk CW tariffs), and errors in predicted utility values were calculated. The average MAE was 0.098 ± 0.022, ranging from 0.063 to 0.142, and the average MSE was 0.020 ± 0.008 ranging from 0.008 to 0.042 (Table 5).

**Table 5:** Error measurement for predicted utility values based on OKS in the external validation sample using different countries value sets

Country	Type of value set	MAE			MSE whole external validation sample(*n* = 115, 100%)
		Whole external validation sample	Utility ≥ median	Utility < median	
		(*n* = 115, 100%)	(*n* = 58, 50.43%)	(*n* = 57, 49.57%)	
Canada	VT	0.076 (0.072–0.085)	0.043 (0.029–0.049)	0.109 (0.101–0.128)	0.011 (0.010–0.013)
China	VT	0.097 (0.086–0.098)	0.072 (0.058–0.091)	0.123 (0.109–0.134)	0.017 (0.014–0.017)
Denmark	VT	0.127 (0.127–0.148)	0.076 (0.077–0.078)	0.178 (0.165–0.179)	0.035 (0.036–0.045)
Egypt	VT	0.134 (0.118–0.157)	0.089 (0.072–0.098)	0.180 (0.166–0.173)	0.033 (0.027–0.044)
England	VT	0.092 (0.091–0.096)	0.064 (0.066–0.075)	0.121 (0.097–0.122)	0.016 (0.015–0.018)
Ethiopia	VT	0.096 (0.093–0.099)	0.042 (0.037–0.049)	0.152 (0.156–0.182)	0.024 (0.022–0.026)
France	VT	0.087 (0.084–0.091)	0.031 (0.032–0.035)	0.143 (0.151–0.162)	0.020 (0.018–0.024)
Germany	VT	0.101 (0.095–0.111)	0.043 (0.036–0.043)	0.160 (0.155–0.178)	0.026 (0.023–0.033)
HongKong	VT	0.128 (0.126–0.128)	0.094 (0.079–0.098)	0.163 (0.164–0.172)	0.027 (0.028–0.028)
Hungary	VT	0.107 (0.109–0.119)	0.049 (0.046–0.051)	0.166 (0.165–0.170)	0.025 (0.025–0.027)
Indonesia	VT	0.125 (0.110–0.125)	0.097 (0.097–0.100)	0.153 (0.136–0.172)	0.025 (0.022–0.025)
Ireland	VT	0.142 (0.139–0.186)	0.088 (0.081–0.106)	0.197 (0.161–0.216)	0.042 (0.041–0.068)
Japan	VT	0.077 (0.066–0.092)	0.078 (0.071–0.083)	0.076 (0.069–0.077)	0.010 (0.008–0.013)
Malaysia	VT	0.095 (0.078–0.099)	0.069 (0.059–0.073)	0.122 (0.103–0.117)	0.016 (0.012–0.017)
Netherlands	VT	0.110 (0.108–0.123)	0.073 (0.065–0.078)	0.148 (0.142–0.160)	0.024 (0.022–0.030)
Peru_cTTO	VT	0.137 (0.137–0.146)	0.110 (0.092–0.117)	0.165 (0.140–0.182)	0.031 (0.035–0.036)
Peru_DCE	VT	0.072 (0.073–0.075)	0.047 (0.040–0.049)	0.098 (0.087–0.113)	0.010 (0.010–0.011)
Poland	VT	0.081 (0.064–0.113)	0.024 (0.016–0.025)	0.140 (0.118–0.156)	0.023 (0.015–0.042)
Portugal	VT	0.092 (0.093–0.104)	0.051 (0.043–0.054)	0.134 (0.123–0.147)	0.018 (0.018–0.023)
SouthKorea	VT	0.070 (0.061–0.071)	0.050 (0.044–0.050)	0.090 (0.083–0.101)	0.009 (0.007–0.009)
Spain	VT	0.099 (0.091–0.102)	0.076 (0.072–0.094)	0.122 (0.104–0.127)	0.018 (0.016–0.018)
Sweden	VT	0.063 (0.062–0.082)	0.045 (0.033–0.048)	0.082 (0.075–0.084)	0.008 (0.007–0.012)
Taiwan	VT	0.140 (0.118–0.158)	0.105 (0.093–0.105)	0.176 (0.159–0.205)	0.035 (0.025–0.039)
Thailand	VT	0.089 (0.074–0.087)	0.060 (0.056–0.076)	0.119 (0.112–0.144)	0.015 (0.011–0.015)
Uruguay	VT	0.066 (0.060–0.072)	0.029 (0.024–0.030)	0.104 (0.101–0.122)	0.011 (0.008–0.012)
USA	VT	0.114 (0.110–0.125)	0.077 (0.067–0.083)	0.152 (0.135–0.148)	0.024 (0.022–0.026)
Vietnam	VT	0.095 (0.090–0.110)	0.071 (0.058–0.069)	0.121 (0.122–0.133)	0.017 (0.016–0.021)
Denmark	CW	0.094 (0.090–0.102)	0.061 (0.062–0.069)	0.127 (0.112–0.144)	0.021 (0.018–0.025)
France	CW	0.121 (0.102–0.130)	0.092 (0.093–0.097)	0.150 (0.130–0.156)	0.023 (0.020–0.025)
Germany	CW	0.082 (0.080–0.091)	0.033 (0.038–0.045)	0.131 (0.130–0.154)	0.018 (0.015–0.021)
Japan	CW	0.072 (0.061–0.072)	0.072 (0.067–0.078)	0.073 (0.060–0.075)	0.012 (0.008–0.012)
Netherlands	CW	0.098 (0.085–0.114)	0.068 (0.060–0.085)	0.129 (0.113–0.135)	0.020 (0.016–0.028)
Russia	CW	0.080 (0.072–0.096)	0.038 (0.037–0.048)	0.123 (0.103–0.130)	0.019 (0.014–0.031)
Spain	CW	0.110 (0.093–0.112)	0.062 (0.051–0.066)	0.159 (0.156–0.159)	0.025 (0.020–0.027)
Thailand	CW	0.099 (0.088–0.109)	0.081 (0.081–0.086)	0.118 (0.097–0.132)	0.019 (0.016–0.022)
UK	CW	0.106 (0.096–0.117)	0.064 (0.047–0.079)	0.148 (0.120–0.157)	0.024 (0.020–0.025)
USA	CW	0.076 (0.074–0.079)	0.048 (0.040–0.064)	0.104 (0.098–0.128)	0.012 (0.012–0.013)
Zimbabwe	CW	0.064 (0.059–0.067)	0.037 (0.033–0.040)	0.092 (0.073–0.090)	0.009 (0.007–0.010)
Mean		0.098	0.063	0.133	0.02
SD		0.022	0.022	0.03	0.008
Min		0.063	0.024	0.073	0.008
Max		0.142	0.11	0.197	0.042

*MAE* mean absolute error; *MSE* mean squared error; *VT* valuation technique; *CW* crosswalk method

95% confidence intervals based on bootstrap percentiles were provided in parentheses

**Box 1:** Structures of model classes used to derive the mapping algorithm for the EQ-5D-5L

1. Cumulative model (CM) It predicts the cumulative probability of an observation being at or below a given level on the outcome. It assumes that ratings originate from the categorization of a latent continuous variable. We varied the structure of CM by modifying the following hyperparameters: *a. Parallel curves or not.* With parallel curves, predictors have the same coefficients across different levels of the outcome. With non-parallel curves, predictors were allowed to have different coefficients *b. Link functions*. Five-link functions were tried to transform the cumulative probability (*p*) to a continuous unbounded scale and can be modeled using ordinal least square regression. They were $logit (p)=\mathrm{log}(\frac{p}{1-p})$;$probit (p)={\varphi }^{-1}(p)$;$cauchit\left(p\right)=\mathrm{tan}(\pi *\left(p-0.5\right))$; $cloglog=\mathrm{ln}(-ln\left(1-p\right))$ and $logc=-\mathrm{ln}(1-p)$ 2. Penalized regression model [27, 38, 39] It fits CM that is penalized for having too many variables in the model. Imposing a penalty reduces the coefficient values; thus, the less contributive predictors have a coefficient close to or equal zero. We varied the structure of penalized regression model by modifying the following hyperparameters: *a. Penalty term (*$\alpha )$*.* We set $\alpha =0$ if penalty was applied to the sum of squared coefficients (Ridge penalized regression), and $\alpha =1$ if penalty was applied to the sum of absolute coefficients (LASSO penalized regression) *b. Criteria used to select the magnitude of penalty.* AIC or BIC *c. Link functions.* Four link functions were used: $logit (p)=\mathrm{log}(\frac{p}{1-p})$;$probit (p)={\varphi }^{-1}(p)$;$cauchit\left(p\right)=\mathrm{tan}(\pi *\left(p-0.5\right))$; and $cloglog=\mathrm{ln}(-ln\left(1-p\right))$ 3. Ordinal CART CART [18] produces a tree to predict both linear and nominal outcomes. It is built-in splitting and pruning. With splitting, the data is partitioned into smaller subsets to minimize impurity in the new subsets as measured by Gini's index. Splitting continues till final homogeneous subsets; however, they might consist of a few similar data points. At this stage, the model predicts the estimation data perfectly, but might not predict a new data point well (overfitting). To avoid this, the tree is pruned back to the point of the least cross-validated overall misclassification We used a modified approach of CART, where a score is assigned to the ordered categories of the outcome [22]. This allows to assign a cost of misclassification; The larger the distance between the actual and predicted levels, the higher the weight given to the misclassification. We varied the structure of produced tree by modifying the following hyperparameters: *a. Cost of misclassification in the generalized Gini index* was calculated in absolute or quadratic terms *b. Complexity Parameter (CP)* is the minimum improvement needed to split at each node. If the split doesn't yield at least that much benefit (the value of cp), the split does not take place. We tried 20 randomly selected values for CP *c. The cross-validated overall misclassification* (used to determine pruning) was measured using: Misclassification error rate, all misclassifications were given same weight Misclassification cost rate, different weights were given to different misclassifications 4. Ordinal forests (OF) Random forest (RF) [17] is a flexible machine-learning algorithm to predict linear and nominal outcomes. It builds multiple decision trees and merges them to produce an accurate and stable prediction. For every tree, it selects a random number of participants and predictors We used a modified version of RF [22, 32]. It translates ordinal levels into scores, but instead of using a fixed score set, it optimizes them. It tries different score sets and builds a small forest to estimate the expected predictive performance of each set. The optimum score set (that achieved the highest predictions using small forests) is used to build the final OF We varied the structure of the OF by modifying the following hyperparameters: *a. Number of score sets tried before the approximation of the optimal score set* maybe 50, 100, or 150 sets *b. Number of trees in the smaller forests* maybe 50, 100, or 150 trees
*c. Number of trees in the final OF using the optimized score set* maybe 200, 400, or 600 final trees

**Box 2:** Structure of the different sets of OKS questions used to feed the models to derive the mapping algorithm for the EQ-5D-5L

1. All the 12 OKS questions as predictors 2. RFE-based important predictors are a subset of OKS questions determined by recursive feature elimination (RFE). RFE fits a random forest model with $5\times 5$-fold cross-validation to recursively eliminate predictors that were not required to build an accurate model [11] 3. Model-based important predictors are a subset of OKS questions which is most relevant to prediction as determined by a built-in algorithm within every model class 4. Pre-processed predictors:
The 12 OKS questions were scaled and centered. Then, principal components (Explaining 90% of the variance in OKS questions) were extracted using principal component analysis (PCA)

The developed algorithms' accuracies vary between countries and tariff types. Sweden and South Korea had the smallest MSEs in utility estimated using the valuation technique tariff, while Ireland, Denmark, and Taiwan had the highest. In utilities estimated using crosswalk tariffs, Zimbabwe, Japan, and the United States exhibited the smallest MSEs. The largest MSEs were in Spain and the United Kingdom.

We compared the MAE between utilities above and below the median estimated utility to assess the fit of models in patients with better and worse utilities (Table 5). The MAE was less than 0.20 in both groups.

## Discussion

The majority of literature evaluating osteoarthritis treatment technologies utilized OKS. The mapping of OKS to EQ-5D-5L allows this literature to be utilized in economic evaluations. The application of response mapping, in which we map onto EQ-5D-5L domains as opposed to the utility index, affords an international advantage. Only one Spanish study [31] mapped OKS to EQ-5D-5L; however, response mapping between both instruments was disregarded; consequently, the mapping algorithm will only aid economic evaluation in Spanish contexts.

Another study [20] developed a response mapping algorithm from OKS with satisfactory prediction accuracy; however, it mapped it to the three level EQ-5D-3L rather than the five level one.

Similar to others [20, 31], we found sufficient conceptual overlap between EQ-5D-5L domains and OKS questions. As Dakin et al. (2013) we found that all OKS and EQ-5D-5L questions loaded into a single principal component; while, variance explained by our component (66%) was higher than theirs (40% for pre-operative sample and 54% for post-operative).

We developed a mapping algorithm that predicts EQ-5D- 5L utility based on OKS responses; model performance was better than the model developed using Spanish tariff [31], where the lowest MAEs obtained using GLM and Breg models, were 0.1127 (0.1014–0.1239) and 0.1141 (0.1031–0.1251). Our MAE was 0.099 (0.091–0.102) using Spain VT value set and 0.110 (0.093–0.112) using Spain CW value set. Although prediction accuracy varied with tariff, our algorithm gave accurate predictions of utilities in the external validation sample using the EQ-5D tariffs (maximum MSE = 0.042).

Models predicting mobility, self-care, usual activity as well as pain/discomfort outperform that predicting anxiety/depression. Because OKS includes questions related to mobility, self-care, usual activities, and pain. Meanwhile, no questions ask about psychological symptoms. Nonetheless, OKS improved the accuracy of predicting anxiety/depression from 30% to 43.5% in the estimation sample and 35.7% in the external validation sample, probably as pain and poor knee function contribute to some of the observed anxiety/depression.

As our sample included patients with comorbidities, KO whether indicated or not for TKR, the developed algorithm is likely to increase the range to which it can be applied. However, its performance in dissimilar populations is unknown.

The response mapping model had the best accuracy in prediction of EQ-5D response levels from OKS responses in UK [20]. Therefore, it was our target method. In addition to producing more accurate predictions in this study, response mapping models do not need to deal with not normal utility distributions. Furthermore, while direct mapping models must be developed for specific tariffs, response mapping algorithms can be applied to any five-level EQ-5D available tariff now or in the future [24]. Response mapping gives rich insights into the relationship between the two instruments. For instance, predicting the proportion of patients with different levels in each domain.

Despite all benefits of response mapping, the belief of the need for a large sample size prevented it from being conducted on many occasions [1, 20, 25, 37]. A recent article [35] provided practical guidance for calculating the sample size required for the development of prediction models with continuous, binary, and time-to-event outcomes. In case of ordinal outcomes, one might think that they could follow the suggested method for a binary outcome model. They would calculate the required minimum sample size for each 2 outcome levels and use the highest minimal sample size. If any of the levels is rare, the estimated sample size will be very high. As patients with L5 in any domain are usually rare, it was thought that very large sample size is needed for response mapping. However, we believe this is applicable when the levels of the ordinal outcomes are separate. In this case, the model predicts the probability of falling into one of two adjacent categories, e.g., the probability of L1 vs L2, L2 vs L3, etc. While, if the ordinal outcome is based on a categorization of a continuous latent variable, the model predicts the cumulative probability (probability of falling at or below a particular point), e.g., probability of being in L3 or more vs being in lower levels (L1 & L2). The availability of two versions of the EQ-5D, where each domain might be categorized into 3 (3L) or five (5L) levels is sufficient theoretical evidence for assuming that the ordinal domains are based on latent continuous variables. Another empirical evidence from the current study is that the optimum model for pain/discomfort contained a single set of coefficients to predict all levels of the outcome (parallel curves).

Another argument against the need for large sample size is the effect size. The larger the effect size the smaller the required sample size [12]. In the current study, the correlation between total OKS and EQ-5D domains was high indicating a large effect size.

While the impact of rare events on estimating the sample size is large, their impact on the overall accuracy of the developed model is small due to the following:

First, the events are rare, thus, their contribution to the overall accuracy will be small. Second, with the use of cumulative ordinal regression, usually, these events are predicted at a closer level. Therefore their accurate prediction which requires a large sample size is of little value.

Due to the aforementioned reasons, some data scientists tend to believe that there are no shortcuts to say if we have enough data. The only way would be to try a sample size and build models [13]. One indication of achieving a sufficient sample size is the consensus on the model accuracy. In the current study, the coefficients of variation (CV) of cross-validated model accuracy to judge the consensus of the model accuracies were all below 10%.

Another problem that might emerge with small sample size is overfitting. Overfitting is a condition where a statistical model captures the random error in the data as well as the relationships between variables. As consequence, the predictive performance and the generalization ability of the model will be degraded [16, 34]. To avoid overfitting the following approaches were taken:

1. Selection of important predictors
2. Cross-validation
3. Penalization in penalized ordinal regression
4. Pruning in O-CART
5. Limiting the number of trees in the final OF

### Strengths

The use of caret package and cross-validation technique allowed for trying four classes of machine learning models for ordinal outcomes (Cumulative Probability Model for Ordinal Data, Penalized Ordinal Regression, CART, and Ordinal random forest). Tuning models' hyperparameters permitted to proceed with 133 different model structures. The use of four different sets of predictors per every model structure increased the number of models tried to 532. Machine learning in mapping was introduced by one study which used a deep neural network (DNN) in mapping from MacNew Heart Disease Health-related Quality of Life questionnaire (MacNew) onto country-specific EQ-5D-5L utility scores[23]. While this study mapped to the utility index (direct mapping), our study introduced the use of machine learning in response mapping.

Another strength is that we assessed the uncertainty around the estimated MAE using bootstrapping which does not depend on assumptions.

### Limitations

Although machine learning algorithms result in accurate predictions using small sample size, they act as black boxes where the process of prediction is not as clear as regression analysis with known coefficients.

Furthermore, mapping is not a substitute for including the EQ-5D in future studies and does not overcome the limitations of either instrument [30].

## Conclusions

The current study derived the mapping algorithm from OKS onto the five domains of EQ-5D-5L. With available EQ-5D-5L utility values, utility scores can be calculated, and the latter enables the estimation of QALYs in an economic evaluation. A machine learning approach presents a promising alternative in the mapping literature that warrants further exploration.

## Supplementary Information

**Additional file 1**. **Appendix 1:** EQ-5D-5L in estimation sample. **Appendix 2:** EQ-5D-5L in external validation sample. **Appendix 3:** Correlation among between domains of the EQ-5D-5L and questions of OKS. **Appendix 4:** Distribution of EQ-5D-5 L utility. **Appendix 5:** Characteristics of the Estimation sample. **Appendix 6:** Characteristics of the External validation sample. **Appendix 7:** Description of EQ-5D-5L Utility and Total OKS Score. **Appendix 8:** Mobility RFE important predictors. **Appendix 9:** Choosing of best model of Mobility. **Appendix 10:** Self-Care RFE important predictors. **Appendix 11:** Choosing of best model of Self-Care. **Appendix 12:** Usual Activities RFE important predictors. **Appendix 13:** Choosing of best model of Usual Activities. **Appendix 14:** Pain/Discomfort RFE important predictors. **Appendix 15:** Choosing of best model of Pain/Discomfort. **Appendix 16:** Anxiety/Depression RFE important predictors. **Appendix 17:** Choosing of best model of Anxiety/Depression. **Appendix 18:** Rankings of OKS questions across the five domains of EQ-5D-5L by recursive feature eliminations in the Estimation sample. **Appendix 19:** Model evaluation in whole external validation sample.

## Data Availability

The data that support the findings of this study are available from the corresponding author, Fawaz H, upon reasonable request.

## Abbreviations

AD: Anxiety/depression domain
CART: Classification and regression trees
CM: Cumulative model
CP: Complexity parameter
CW: Crosswalk method
DALYs: Disability-adjusted life years
EQ-5D-5L: EuroQoL five domains five levels
KOA: Knee osteoarthritis
L: Level of the domain of EQ-5D-5L
MAE: Mean absolute error
MO: Mobility domain
MSE: Mean squared error
OA: Osteoarthritis
O-CART: Ordinal classification and regression trees
OF: Ordinal forests
OKS: Oxford Knee Score
OLS: Ordinary least squares
PCA: Principal component analysis
PD: Pain/discomfort domain
PROMs: Patient-reported outcome measures
QALYs: Quality-adjusted life-year
QoL: Quality of life
RF: Random forest
RFE: Recursive feature elimination
SC: Self-care domain
TKA: Total knee arthroplasty
UA: Usual activities domain
VAS: A visual analogue scale
VT: Valuation technique
